# Eucalyptus oil reduces allergic reactions and suppresses mast cell degranulation by downregulating IgE-FcεRI signalling

**DOI:** 10.1038/s41598-020-77039-5

**Published:** 2020-12-01

**Authors:** Tomoya Nakamura, Naoki Yoshida, Yu Yamanoi, Akira Honryo, Hiroyuki Tomita, Hiroki Kuwabara, Yoshihiko Kojima

**Affiliations:** Department of R&D Center, Ikeda Mohando Co., Ltd, 16 Jinden, Kamiichi, Nakaniikawa, Toyama 930-0394 Japan

**Keywords:** Molecular biology, Health care, Cell biology, Cell signalling, Immunology, Cytokines, Chemokines, Interleukins, Medical research, Drug development, Diseases, Skin diseases

## Abstract

Eucalyptus oil has been used since ancient times for its bactericidal, anti-inflammatory, analgesic and sedative effects. In recent years, the action of Eucalyptus oil has been scientifically proven, and there have been reports that Eucalyptus oil suppresses the production of chemokines, cytokines and lipid mediators in basophils, alveolar macrophages and monocytes. Based on this information, we aimed to verify whether Eucalyptus oil can be used for allergic dermatitis, the incidence of which has been increasing among human skin diseases. This effect was verified using a mouse IgE-mediated local allergic model. In conclusion, topical application of Eucalyptus oil suppressed oedema and vascular permeability enhancement due to IgE-mediated allergic on the skin. In addition, we also verified the degranuration of mast cells, which is a part of its action, and examined whether 1,8-cineole, which is the main component of Eucalyptus oil, suppresses the phosphorylation of PLCγ and p38 directly or indirectly. 1,8-cineole was found to suppress degranulation of mast cells.

## Introduction

Eucalyptus oil has been used by Australian natives as a remedy for wounds and inflammation^1,2^. Essential oils obtained from Eucalyptus leaves are said to have anti-inflammatory, analgesic, sedative and bactericidal effects, and they are used in medicines and aromatherapy^3^. In the UK, oil extracted from *Eucalyptus globulus* is used to treat airway inflammation and various symptoms of rheumatism. In Japan, the essential oil obtained via steam distillation of the leaves of *Eucalyptus globulus Labillardière* or other closely related plants (Myrtaceae) is used as an anti-inflammatory and analgesic. Clinical studies confirmed the anti-inflammatory effects of Eucalyptus oil in patients with asthma, such as alleviating sinusitis symptoms and preventing the worsening of chronic obstructive pulmonary disease^4–7^. A comprehensive report on plant oil using basophils has reported the inhibitory effect of Eucalyptus oil on IgE-mediated basophil activation^8^. It is suggested that Eucalyptus oil suppresses inflammation by directly acting on immune cells such as macrophages and monocytes^9^, as supported by several studies. Specifically, Eucalyptus oil was demonstrated to inhibit lipopolysaccharide (LPS)-induced nitric oxide production in mouse macrophage cell lines^10^ improve the acute inflammatory response of the lungs in a mouse acute lung injury model^11^ and inhibit active oxygen release by cultured alveolar macrophages from patients with chronic obstructive pulmonary disease^12^. Additionally, 1,8-cineole, the main component of Eucalyptus oil, blocked arachidonic acid metabolism in blood monocytes from patients with asthma^13^ and inhibited LPS-induced IL-1β production by human monocytes^14^. In recent years, it has also been reported that eucalyptol exerts anti-inflammatory effects in mice through effects on TRPM8 channels^15^.

Eucalyptus oil was shown to have an analgesic effect in the formalin and carrageenan paw oedema test despite being applied topically^16,17^. As a bath agent, it was reported to improve the skin symptoms of patients with atopic dermatitis^18^.

Therefore, based on this information, we aimed to verify whether or not Eucalyptus oil can be used for allergic dermatitis, the incidence of which has been increasing among human skin diseases.

In this study, we first evaluated whether Eucalyptus oil suppressed inflammation in an IgE-mediated local allergic model, a model of inflammatory allergic disease caused by mast cell activation^19–21^. We confirmed that the anti-inflammatory effect of Eucalyptus oil in wild-type mice was attributable to the suppression of mast cell activation based on the findings in mast cell deficient mice. Because Eucalyptus oil suppressed mast cell degranulation, the mechanism of action was also examined using an in vitro cell assay system and bone-marrow-derived mast cells (BMMCs), which revealed that Eucalyptus oil and its main component 1,8-cineole suppressed mast cell degranulation and alleviated allergic dermatitis, supporting their potential as crude medicines for relieving allergic dermatitis.

## Results

### Eucalyptus oil suppresses IgE-mediated local allergic inflammation in mice

**Figure 1:**
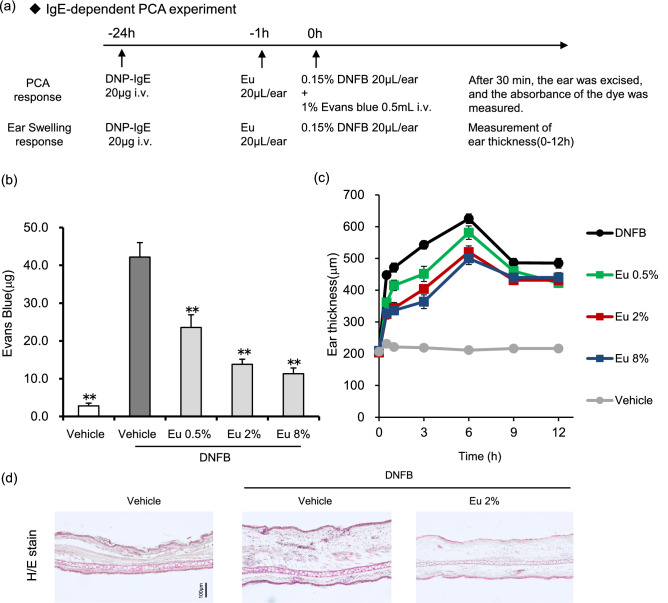

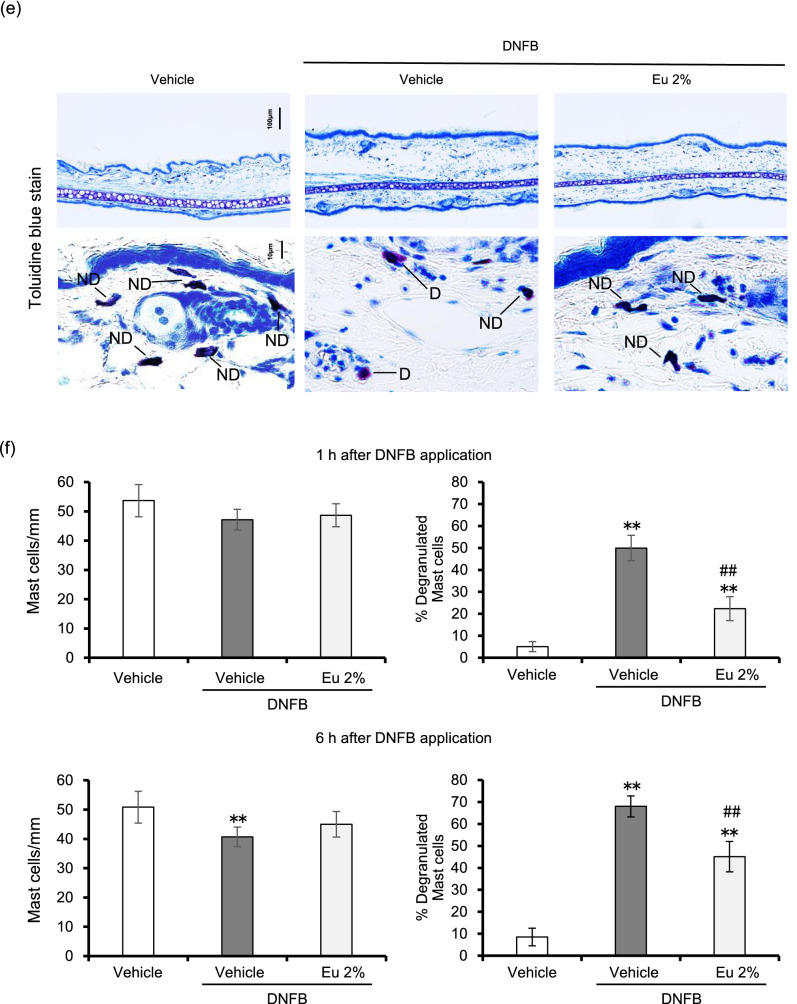
Eucalyptus oil (Eu) suppresses passive cutaneous anaphylaxis (PCA) in a mouse model. (**a**) Schedule of treatment and experimental procedures for the PCA and ear swelling response tests. (**b**) Anti-DNP-IgE was injected intravenously 24 h before DNFB application. One hour before DNFB application, Eucalyptus oil or vehicle (3:1 acetone/olive oil) was applied to the inside of each ear of all animals. DNFB was applied to the outside of the ear, followed by an immediate injection of Evans blue dye. Thirty minutes later, the ears were excised, and the absorbance of the dye was measured. Data are presented as the mean ± SEM (N = 8/group). The statistical significance of the differences was assessed using Dunnett’s test (**P < 0.01 versus vehicle-DNFB). (**c**) The time course of ear oedema was investigated. Ear thickness was measured using a thickness gauge. Data are presented as the mean ± SEM (N = 8/group). (**d**) Haematoxylin and eosin staining of ear tissue 6 h after DNFB application. (**e**,**f**) Toluidine blue staining of ear tissue 1 or 6 h after DNFB application. (**e**) Tissue sections were mast cells obtained from ear tissue collected 6 h after the application of DNFB. Mast cells were identified in tissue sections by their characteristic granular, deep blue-purple metachromatic appearance against blue orthochromatic background tissue. *ND* no degranulation, *D* degranulation. (**f**) Left panel shows the number of cutaneous mast cells at the dermal/epidermal (D/E) junction. Right panel shows the percentage of degranulated mast cells. Each bar is mean ± SEM (N = 6/group) of 12 fields of views. Statistical significance of the differences was assessed using the Tukey–Kramer test (**P < 0.01 versus vehicle, ##P < 0.01 versus vehicle-DNFB).

To determine whether Eucalyptus oil can suppress mast cell degranulation, we tested its effects using passive cutaneous anaphylaxis (PCA) responses in mice. PCA is a local skin allergic reaction resulting from hyperpermeability and plasma extravasation following allergen exposure, and it has been used as an animal model for IgE-mediated allergic reactions^22,23^. We evaluated whether Eucalyptus oil can suppress IgE-mediated allergic reactions by vascular hyperpermeability and local ear oedema by assessing the leakage of Evans blue dye (Fig. 1).

In addition, prior to the Eucalyptus oil test, it was verified whether the inhibitory effect can be detected, using sodium cromoglycate, which has an inhibitory effect on mast cell degranulation (Supplementary Fig. 2).

Eucalyptus oil significantly suppressed the amount of dye leaking into the tissue compared with the effects of the vehicle-DNFB. Eucalyptus oil also inhibited dye leakage in a dose-dependent manner (Fig. 1b). Meanwhile, oedema was induced in the ears of mice following DNFB application, peaking at 6 h, and Eucalyptus oil inhibited this oedema in a dose-dependent manner (Fig. 1c).

Haematoxylin and eosin (H&E) staining of the auricle was performed 6 h after DNFB application. As a result, swelling of the dermal tissue, thickening of the stratum corneum and infiltration of inflammatory cells into the dermal layer via DNFB application were noticed, and these findings were suppressed by Eucalyptus oil (Fig. 1d).

Toluidine blue staining was performed to confirm that Eucalyptus oil suppressed mast cell degranulation after DNFB application. The number of degranulated mast cells was quantified by counting the mast cells in the image of skin tissue^24,25^.

Almost no degranulation of mast cells was observed in the vehicle. Vehicle-DNFB degranulated 50% of mast cells after 1 h and 68% after 6 h. Furthermore, after 6 h, the number of mast cells confirmed by toluidine blue staining reduced.

In the group to which the Eucalyptus oil was applied, a significant suppression of mast cell degranulation rate was observed 1 h after the application of DNFB as compared with that observed after the application of vehicle-DNFB. A significant inhibitory effect was observed even 6 h after DNFB application; however, the inhibitory effect observed 1 h after DNFB application was weaker than that observed 6 h after DNFB application (Fig. 1e,f).

These results revealed that Eucalyptus oil suppressed PCA induced by anti-DNP-IgE antibody exposure in mice. It is known that mast cell degranulation is strongly involved in the very early phase of ear oedema 1 h after reaction caused by intravenous administration of anti-DNP IgE antibody and subsequent application of DNFB, and that migration of inflammatory cells is involved in the early phase of ear oedema^26,27^. The ear oedema suppression effect of Eucalyptus oil has been observed from a relatively early stage and this effect gradually weakens after 3 h. As a result of the inhibition of mast cell degranulation by toluidine blue staining, it was clarified that Eucalyptus oil plays a role in mast cell degranulation inhibition.

However, the inflammation caused by anti-DNP-IgE antibodies also involves inflammatory cells other than mast cells and therefore we aimed to clarify how Eucalyptus oil affects mast cell deficient mice^27,28^.

### The inhibitory effect of Eucalyptus oil was not observed in mice lacking mast cells

**Figure 2:**
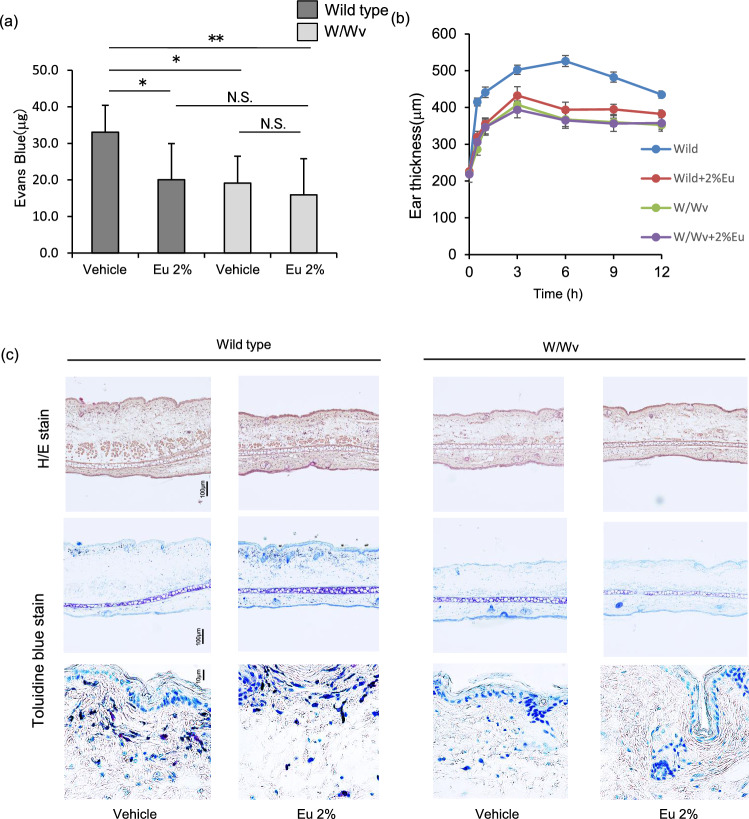
Inhibitory effect of Eucalyptus oil (Eu) on passive cutaneous anaphylaxis (PCA) in mast celldeficient mice. (**a**) Effect of Eucalyptus oil on Evans blue dye leakage in wild-type and W/Wv mice. One hour before DNFB application, Eucalyptus oil or vehicle (3:1 acetone/olive oil) was applied to the inside of the ears of wild-type and W/Wv mice sensitised with anti-DNP-IgE. DNFB was applied to the outside of the ear, which was immediately injected intravenously with Evans blue dye. Thirty minutes later, the ears were excised, and the absorbance of the dye was measured. Data are presented as the mean ± SEM (N = 8). The statistical significance of the differences was assessed using Tukey–Kramer test (**P < 0.01, *P < 0.05 versus vehicle). (**b**) Time-dependent changes in the suppression of pinna oedema by Eucalyptus oil in wild-type and W/Wv mice. Ear thickness was measured using a thickness gauge. Data are presented as the mean ± SEM (N = 8). (**c**) Haematoxylin and eosin-stained and toluidine blue-stained images of ear tissue from Eucalyptus-oil-treated wild-type and W/Wv mice 6 h after DNFB application.

W/Wv mice are known to lack mast cells because of the lack of mast cell progenitor cells, and they are widely used to confirm the physiological roles of mast cells^28–30^. We measured the ear thickness by PCA reaction and analysed the inhibitory effect of Eucalyptus oil on the increase of vascular permeability. In wild-type mice, Eucalyptus oil significantly suppressed dye leakage into the tissue compared with the effects of the vehicle. In W/Wv mice deficient in mast cells, both vehicle and Eucalyptus oil had significantly less tissue dye leakage than wild-type vehicle application. Also, there was no difference in the dye leakage by vehicle and Eucalyptus oil (Fig. 2a).

DNFB application induced oedema in the ears of wild-type mice, peaking at 6 h in the vehicle (Fig. 2b), and these effects were suppressed by Eucalyptus oil. The vehicle- and Eu-treated W/Wv mice showed increased ear thickness with the application of DNFB, peaking at 3 h, but the increase was obviously smaller in the vehicle-treated wild-type mice (Fig. 2b).

H&E and toluidine blue staining of the auricle 6 h after DNFB application revealed swelling of dermal tissue, thickening of the stratum corneum and infiltration of inflammatory cells into the dermal layer in vehicle-treated wild-type mice, and these effects were suppressed by Eucalyptus oil treatment (Fig. 2c, Supplementary Fig. 3). Vehicle-treated W/Wv mice exhibited less swelling of dermal tissue, thickening of the stratum corneum and infiltration of inflammatory cells into the dermis layer compared with the findings in vehicle-treated wild-type mice, but there was no difference in the findings between vehicle- and Eucalyptus-oil-treated W/Wv mice (Fig. 2c).

Toluidine blue staining of the ear 6 h after DNFB application confirmed mast cell degranulation in vehicle-treated wild-type mice. Fewer mast cells were degranulated in Eucalyptus-oil-treated wild-type mice. Meanwhile, no mast cells were identified in either vehicle- or Eucalyptus-oil-treated W/Wv mice (Fig. 2c).

Predictably, treatment with vehicle and Eucalyptus oil in mast cell deficient W/Wv mice did not differ in terms of the inhibitory effect on PCA responses. However, W/Wv and wild-type mice are known to have no differences in T lymphocytes, B lymphocytes and NK cells^31^, but have low anaemia and red blood cell count due to lack of skin pigment cells^27^. Therefore, detailed thorough examination is needed in the future.

Mast cells play an important role in IgE-mediated hypersensitivity reactions, making them an important target for alleviating allergic symptoms^32^. When mast cells are exposed to antigens, IgE-binding high-affinity IgE receptors (FcεRI) on the plasma membrane are cross-linked, triggering mast cell activation and degranulation. Therefore, an in vitro cell-based assay using BMMCs was performed to investigate the mechanism by which Eucalyptus oil suppresses mast cell degranulation.

### Eucalyptus oil and 1,8-cineole inhibit the degranulation of BMMCs

The effects of Eucalyptus oil and its main component 1,8-cineole on BMMC degranulation were investigated. Anti-DNP-IgE antibody-sensitised BMMCs were treated with Eucalyptus oil or 1,8-cineole and stimulated with DNP-human serum albumin (HSA) as an antigen. The amount of β-hexosaminidase released from the cells was used as a marker of enzymatic activity to evaluate the effects of Eucalyptus oil and 1,8-cineole on mast cell degranulation. β-Hexosaminidase is released together with histamine during mast cell degranulation, and the amount released is generally used as an indicator of degranulation^33,34^.

**Figure 3:**
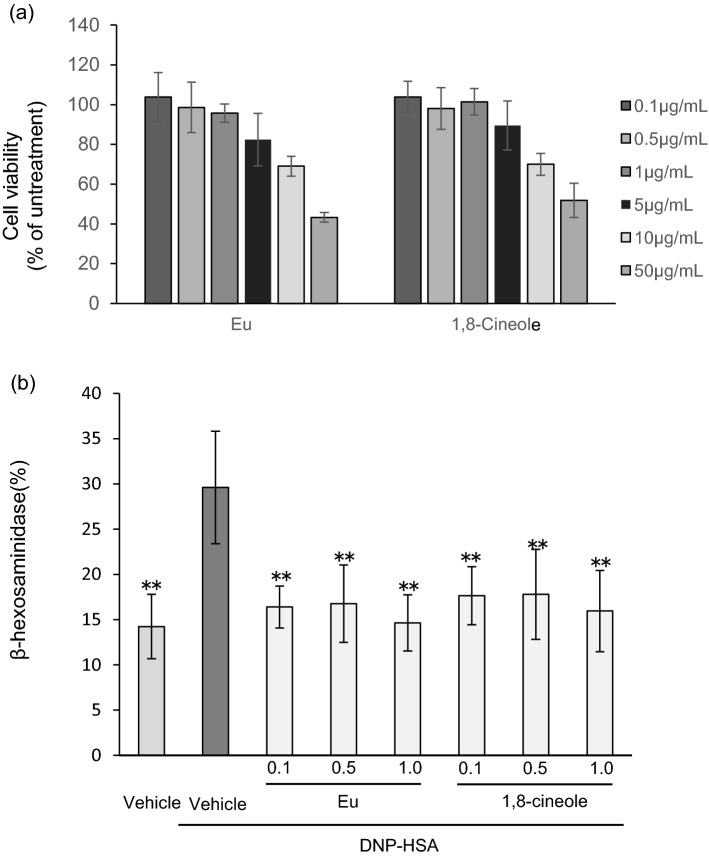
Effect of Eucalyptus oil (Eu) and 1,8-cineole on IgE-induced degranulation in bone-marrow-derived mast cells (BMMCs). (**a**) Effect of Eucalyptus oil and 1,8-cineole on BMMCs viability. Cells were treated with various concentrations of Eucalyptus oil or 1,8-cineole for 24 h. Next, cell viability was measured using the MTT assay. Relative cell viability was calculated by comparing the absorbance in cells treated with Eucalyptus oil or 1,8-cineole with that in cells treated with vehicle (0.1% DMSO). Data are expressed as the mean ± SEM (N = 3). (**b**) Eucalyptus oil or 1,8-cineole was applied to anti-DNP-IgE-sensitised BMMCs immediately after stimulation with DNP-human serum albumin (HSA), and after 1 h, β-hexosaminidase release was determined by comparing the enzymatic activity in BMMCs between treatment with vehicle (0.1% DMSO) and DNP-HSA. Data are expressed as the mean ± SEM (N = 4). Dunnett’s test was used to assess the statistical significance (**P < 0.01versus vehicle-DNP-HSA).

First, the viability of BMMCs following exposure to various concentrations of Eucalyptus oil or 1,8-cineole was examined. Both Eucalyptus oil and 1,8-cineole had no effect on cell viability at concentrations below 1 µg/mL (Fig. 3a). When Eucalyptus oil or 1,8-cineole was applied to BMMCs at a concentration of 0.1, 0.5 or 1 µg/mL, β-hexosaminidase release was significantly suppressed. These results indicated that Eucalyptus oil and 1,8-cineole suppressed mast cell degranulation (Fig. 3b).

### Eucalyptus oil and 1,8-cineole suppress inflammatory chemokine, cytokine and lipid mediator production by BMMCs

**Figure 4:**
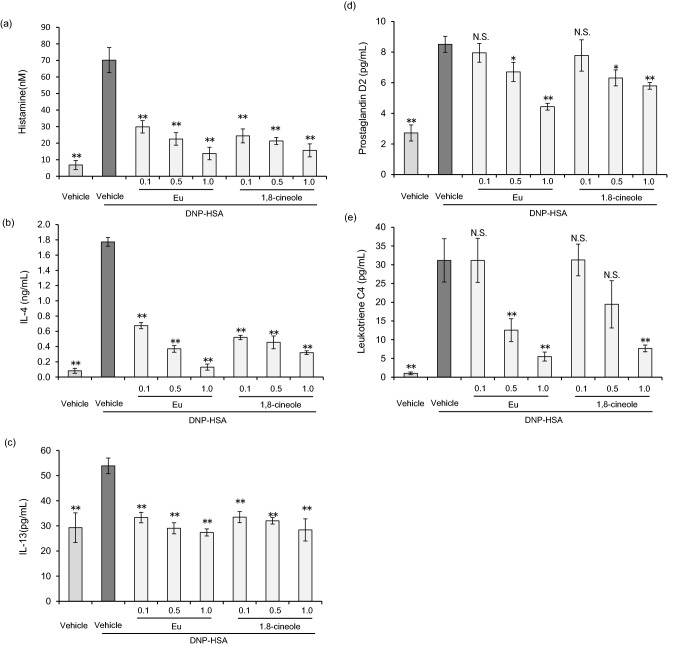
Effects of Eucalyptus oil (Eu) and 1,8-cineole on inflammatory chemokine and cytokine production by bone-marrow-derived mast cells (BMMCs). (**a**) Histamine release was determined after treating anti-DNP-IgE-sensitised BMMCs with Eucalyptus oil and 1,8-cineole. Histamine release in the supernatant was specifically after 30 min of stimulation with DNP-human serum albumin (HSA) via ELISA. Histamine release was calculated relative to that in cells treated with vehicle (0.1% DMSO) and stimulated with DNP-HSA. Data are expressed as the mean ± SEM (N = 4). Dunnett’s test was used to assess the statistical significance (**P < 0.01 versus vehicle-DNP-HSA). (**b**,**c**) Production of IL-4 and IL-13 by anti-DNP-IgE-sensitised BMMCs was determined after treatment with Eucalyptus oil or 1,8-cineole. ELISA was performed after culturing cells with DNP-HSA for 3 h. IL-4 and IL-13 production was calculated relative to that in cells treated with vehicle (0.1% DMSO) and stimulated with DNP-HSA. Data are expressed as the mean ± SEM (N = 4). Dunnett’s test was used to assess the statistical significance (**P < 0.01 versus vehicle-DNP-HSA). (**d**,**e**) Production of prostaglandin D2 and leukotriene C4 by anti-DNP-IgE-sensitised BMMCs was determined after treatment with Eucalyptus oil or 1,8-cineole. ELISA was performed after culturing cells with DNP-HSA for 1 h. Prostaglandin D2 and leukotriene C4 production was calculated relative to that in cells treated with vehicle (0.1% DMSO) and stimulated with DNP-HSA. Data are expressed as the mean ± SEM (N = 4). Dunnett’s test was used to assess the statistical significance (**P < 0.01) of differences between vehicle- and DNP-HSA-treated cells. Dunnett’s test was used to assess the statistical significance (*P < 0.05, **P < 0.01 versus vehicle-DNP-HSA).

Mast cells activated locally by inflammation produce histamine as well as cytokines such as IL-4 and IL-13 and lipid mediators such as prostaglandin and leukotriene C4, thereby promoting allergic inflammation^32,35^. The effects of Eucalyptus oil and 1,8-cineole on the production of histamine, IL-4, IL-13, prostaglandin and leukotriene C4 were investigated via an in vitro assay using BMMCs. When BMMCs sensitised with anti-DNP-IgE were stimulated with the antigen, histamine, IL-4, IL-13, prostaglandin and leukotriene C4 were detected in the supernatant (Fig. 4a–e). When Eucalyptus oil or 1,8-cineole was applied at 0.1, 0.5 or 1 µg/mL, histamine production was significantly inhibited (Fig. 4a), and IL-4 and IL-13 production was also significantly suppressed (Fig. 4b,c). Neither Eucalyptus oil nor 1,8-cineole inhibited the production of prostaglandin and leukotriene C4 at 0.1 g/mL. At a concentration 0.5 µg/mL, their production was suppressed, albeit without significance. At 1 µg/mL, significant suppression was observed (Fig. 4d,e). There was no difference between the inhibitory effects of Eucalyptus oil and 1,8-cineole. Regarding mast cell degranulation, the effects of Eucalyptus oil could be attributable to 1,8-cineole. The inhibitory effects on prostaglandin D2 and leukotriene C4 were weaker than those on histamine, IL-4 and IL-13.

### Eucalyptus oil and 1,8-cineole suppress the increase in intracellular Ca^2+^ concentration in BMMCs

When IgE is cross-linked by the antigen, FcεRI aggregation initiates signalling cascades, leading to the increase in intracellular Ca^2+^  concentration is one of the major signalling pathways for mast cell degranulation^36^. We next investigated the effect of 1,8-cineole on the intracellular Ca^2+^ increase in BMMCs.

**Figure 5:**
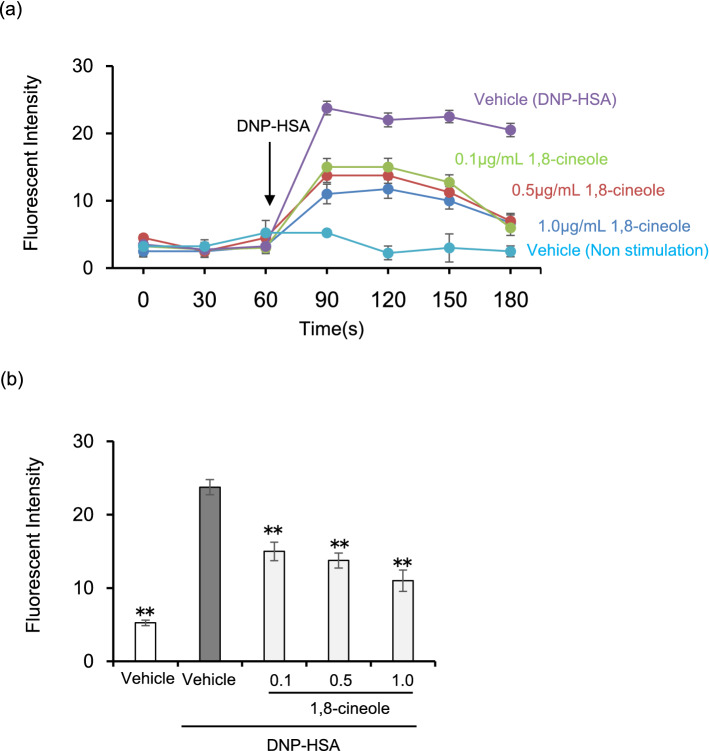
1,8-cineole suppresses the elevation of intracellular Ca^2+^ concentration. BMMCs sensitised with anti-DNP IgE were incubated in medium containing Fluo 4-AM. BMMCs were treated with various concentrations of 1,8-cineole and stimulated with DNP-HSA 60 s after initiating the monitoring of fluorescence intensity. (**a**) Time course of intracellular Ca^2 +^ concentration in BMMCs. Data are displayed as mean ± SEM (N = 5). (**b**) Relative fluorescence intensity 60 s after DNP-HSA stimulation. Data are displayed as mean ± SEM (N = 5). Statistical significance was assessed using Dunnett’s test (** P < 0.01 vs vehicle-DNP-HSA).

Stimulation with DNP-HSA was performed for 60 s after the start of Ca^2+^ measurement. As depicted in Fig. 5a, the fluorescence intensity of Fluo 4 increased immediately after the DNP-HSA stimulation of vehicle-treated cells, whereas the fluorescence intensity of Fluo 4 remained unchanged without DNP-HSA. Fluo 4 fluorescence intensity was significantly suppressed when cells treated with 1,8-cineole were stimulated with DNP-HSA.

Figure 5b shows the results 60 s after DNP-HSA stimulation. Compared with the fluorescence intensity of DNP-HSA-stimulated cells treated with vehicle, there was significant suppression at 0.1, 0.5 and 1.0 μg/mL every concentration of 1,8-cineole, and although no significant difference was detected, a concentration-dependent suppression was observed.

It was found that 1,8-cineole suppressed the intracellular Ca^2+^ increase stimulated by DNP-HSA. Therefore, we next examined the effect of 1,8-cineole on the intracellular signalling cascades of BMMCs other than Ca^2+^.

### 1,8-cineole does not affect Lyn and Syk phosphorylation

Mast cells express FcεRI on their surface, and when IgE is cross-linked by an antigen, the Src family tyrosine kinase Lyn, which is related to FcεRI, is phosphorylated, followed by a series of signal transductions. Then, signs of mast cell activation such as degranulation, cytokine production and lipid mediator production are observed^29,32,37,38^. Therefore, the effects of 1,8-cineole on the phosphorylation status of intracellular signalling proteins were examined via immunoblot analysis.

**Figure 6:**
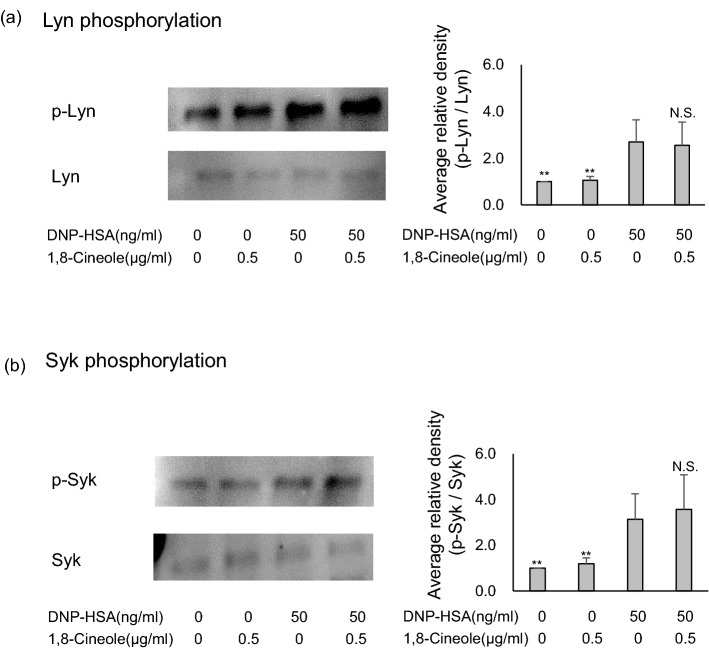
Effect of 1,8-cineole on the phosphorylation of Syk and Lyn. (**a**,**b**) Eucalyptus oil or 1,8-cineole was applied to BMMCs sensitised with anti-DNP-IgE 24 h before stimulation with DNP-human serum albumin, and then after 10 min, cells were lysed, separated by SDS-PAGE and immunoblotted to analyse p-Syk, Syk, p-Lyn and Lyn expression. The left side presents representative examples of blots for each protein, and the right bar graphs present the phosphorylation relative ratios for Syk and Lyn.Phosphorylation ratios were calculated relative to Syk and Lyn expression (each set as 1), and data are presented as the mean ± SEM of 4–6 independent experiments. Dunnett’s test was used to assess the statistical significance (**P < 0.01 versus DNP-HSA). The original blots are shown in the supplementary Fig. 5.

Anti-DNP-IgE antibody-sensitised BMMCs were treated with 0.5 µg/mL 1,8-cineole, stimulated with DNP-HSA as an antigen, lysed and immunoblotted. As a result, the phosphorylation of Syk and Lyn was not suppressed by 1,8-cineole, even when the concentration was increased to as much as 2.0 µg/mL (Fig. 6a,b).

### 1,8-cineole suppresses PLCγ and p38 phosphorylation

**Figure 7:**
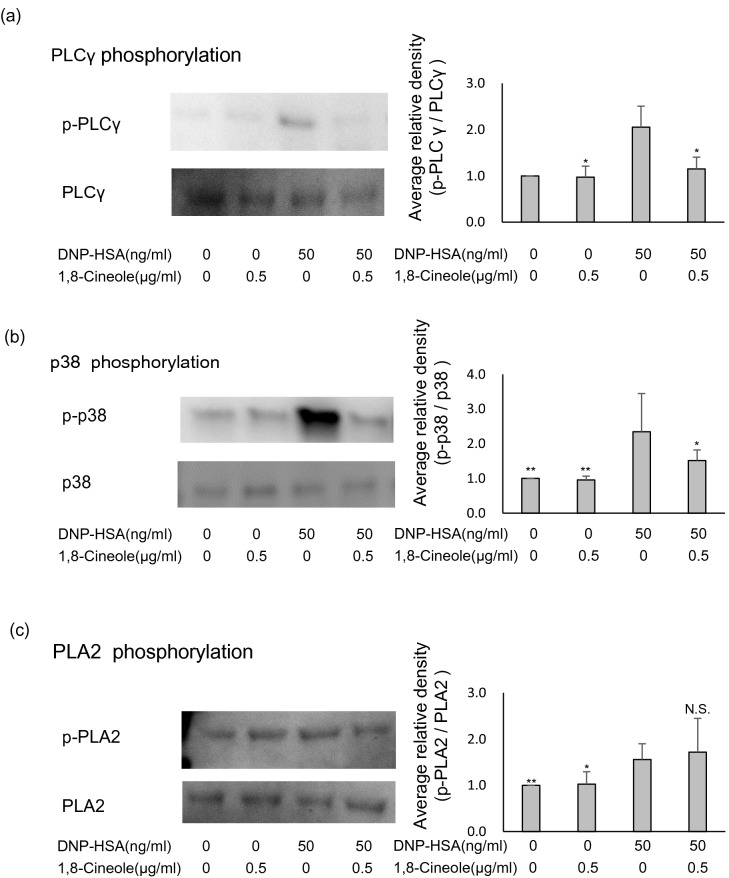
Effect of 1,8-cineole on the phosphorylation of PLCγ, p38 and phospholipase A2 (PLA2). (**a**–**c**) 1,8-cineole was applied to bone-marrow-derived mast cells (BMMCs) after sensitisation with anti-DNP-IgE for 24 h, and cells were subsequently stimulated with DNP-human serum albumin (HSA). After 10 min, the cells were lysed, and SDS-PAGE was performed. Proteins were separated, and p-PLCγ, PLCγ, p-p38, p38, p-PLA2 and PLA2 expression was examined via immunoblotting. The left side presents representative blots for each protein, and the right bar graphs show the relative ratios of PLCγ, p38 and PLA2 phosphorylation. Phosphorylation ratios were calculated relative to PLCγ, p38 and PLA2 expression (each set as 1), and data are presented as the mean ± SEM of 4–6 independent experiments. Dunnett’s test was used to assess the statistical significance (**P < 0.01 versus DNP-HSA). The original blots are shown in the supplementary Figs. 6, 7.

Lyn and Syk phosphorylation was not inhibited by 1,8-cineole. Therefore, we investigated the intracellular signalling cascade downstream of Lyn and Syk. Anti-DNP-IgE antibody-sensitised BMMCs were treated with Eucalyptus oil or 1,8-cineole, stimulated with DNP-HSA as an antigen, lysed and immunoblotted. As a result, PLCγ phosphorylation was significantly suppressed by 0.5 μg/mL 1,8-cineole (Fig. 7a), as was p38 phosphorylation (Fig. 7b). Phosphorylation of phospholipase A2 (PLA2) was not suppressed by 0.5 µg/mL 1,8-cineole(Fig. 7c). The results confirm that 1,8-cineole suppressed PLCγ and p38 phosphorylation.

## Discussion

To confirm the usefulness of Eucalyptus oil before starting this test, we conducted a comparative test with Orange oil,Hinokitiol,L-menthol,dl-camphol, γ-oryzanol, etc., which are plant oils known to have degranulation inhibitory effects on basophils^8,39,40^, and confirmed the potential of Eucalyptus oil (Supplementary Fig. 1). In this study, we aimed to investigate the suppression of the degranulation effect of Eucalyptus oil by topical application and its mechanism of action.

Using an IgE-sensitised allergy mouse model^26,27^, this study revealed that Eucalyptus oil may be effective against IgE-mediated allergic dermatitis. The therapeutic effect was demonstrated even when applied externally to the skin.

In a test in which IgE-sensitised allergy was induced in mast cell deficient mice, Eucalyptus oil had a suppressive effect with a result same as that of the vehicle, and it was speculated that Eucalyptus oil is involved in suppressing the degranulation of mast cells.

Moreover, the release of histamine, which is considered to be caused by degranulation, the production of cytokines such as IL-4 and IL-13, and the production of lipid mediators such as prostaglandin D2 and leukotriene C4 were suppressed in the test using BMMCs.

The incidence of allergic diseases caused by mast cell activation such as bronchial asthma, atopic dermatitis and hay fever has increased in recent years^32,33^. Antihistamines and mast cell stabilisers are commonly used to treat IgE-mediated allergic diseases. Although antihistamines are the most commonly prescribed medications to improve allergic symptoms, they can cause adverse effects such as drowsiness and headaches^41^.

Eucalyptus oil has been reported to induce oedema and peritoneal mast cell degranulation in rats at high concentrations when administered intra-dermally^42^, but no side effects such as sleepiness have been reported. In the present IgE-sensitised allergy model, the Eucalyptus oil was applied transdermally and there were no risks of adverse effects; thus, it can be used as a topical treatment separately or in combination with antihistamines.

In a topical study using 10% Eucalyptus oil, no irritation or sensitisation was noted in 25 subjects^41^. The recommended concentration for external 1,8-cineole application is 20–25% or less according to the Commission E Monograph of Germany and the Canadian Department of Health. Thus, 1,8-cineole is considered a useful treatment for allergic diseases if used topically at a concentration not exceeding 20%.

On the other hand, to clarify the degranulation inhibitory mechanism of Eucalyptus oil in BMMCs, we investigated the increase of intracellular Ca^2+^ concentration in BMMCs using 1,8-cineole, which accounts for 80% of Eucalyptus oil.

Our results showed that 1,8-cineole suppressed the increase of Ca^2+^ in BMMCs. It is known that the increase of Ca^2+^ in BMMCs by the FcεRI aggregation signal is associated with the phosphorylation signal immediately below FcεRI^37,38^. Therefore, we explored whether 1,8-cineole suppresses the phosphorylation of Lyn and syk^43,44^, which play important roles in the signalling cascade immediately below FcεRI. However, 1,8-cineole did not suppress the phosphorylation of Lyn and Syk. This is consistent with the result that PLA2 phosphorylation is not suppressed.

**Figure 8:**
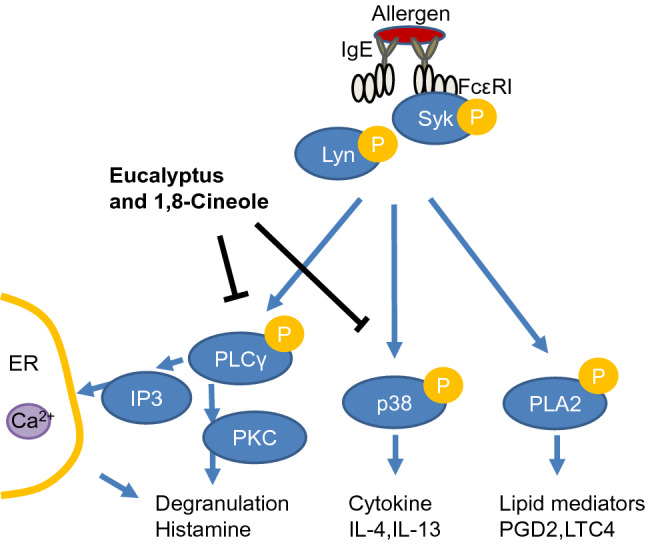
Eucalyptus and 1,8-cineole are putative sites of inducible inhibition of mast cell degranulation.

Next, we confirmed the phosphorylation of PLCγ, p38, and PLA2, which are the downstream signals of Syk. 1,8-cineole suppressed the phosphorylation of PLCγ and p38. PLCγ is known as an upstream proteins of the degranulation-related molecule DAG, PKC and IP3^19,20,38^. 1,8-cineole directly or indirectly suppressed the phosphorylation of PLCγ. Therefore, it was believed that it suppressed the increase of intracellular Ca^2+^ and the release of histamine through degranulation (Fig. 8).

Studies have reported that p38 is located in the upstream of the production of cytokines and chemokines^20,35,38^. We considered that the production of cytokines such as IL-4 and IL-13 was suppressed by directly or indirectly suppressing the phosphorylation of p38 (Fig. 8). However, it is known that the production of cytokines and chemokines involves several factors such as ERK, JNK and AKT^35,38^, and therefore, a more detailed analysis was considered to be necessary.

Lastly, no phosphorylation of PLA2 by 1,8-cineole could be confirmed. This result is inconsistent with the result of PGD2 and LTC4 suppression by ELISA. From the knowledge that PLA2 affects the production of lipid mediators PGD2 and LTC4^34^, we assume that 1,8-cineole inhibits PGD2 and LTC4 production by pathways other than inhibition of PLA2 phosphorylation. However, the details could not be revealed in our survey.

In conclusion, we demonstrated that application of Eucalyptus oil to mice with IgE-mediated allergic dermatitis suppresses vascular hyperpermeability and oedema of the skin. Moreover, focusing on one of these action points, the degranulation of mast cells, 1,8-cineole acts directly or indirectly on the phosphorylation of PLCγ and p-38 to suppress degranulation.

## Materials and methods

### Mice

Female BALB/c mice (Japan SLC, Shizuoka, Japan) and male WBB6F1 + / + (wild-type) and WBB6Ft/J-W/Wv (W/Wv) mice (Japan SLC, Shizuoka, Japan) were used at 6–12 weeks old. The mice were housed in a room under controlled temperature (22 °C ± 2 °C), humidity (50% ± 10%) and light (lights on from 07:00 to 19:00). Food and water were freely available. The study was approved by the Animal Research Committee of Ikedamohando Research Center in accordance with the National Research Council Guidelines.

### Reagents

DNCB, anti-DNP-IgE and DNP-HSA were purchased from Sigma-Aldrich (St. Louis, MO, USA), Eucalyptus oil, procured from Nippon Terpene Chemicals (Tokyo, Japan), conforms with the Japanese Pharmacopoeia, and its main component, 1,8-cineole, accounts for 82.5% of the oil. 1,8-cineole was obtained from Nacalai Tesque (Kyoto, Japan), and dimethyl sulfoxide (DMSO) was acquired from Wako (Osaka, Japan). The following antibodies for immunoblotting were purchased from Cell Signalling Technology (Beverly, MA, USA): anti-Syk, anti-phospho-Syk, anti-Lyn, anti-phospho-Lyn, anti-PLA2, anti-phospho-PLA2, anti-PLCγ, anti-phospho-PLCγ, anti-p38 and anti-phospho-p38. Goat anti-rabbit IgG horseradish peroxidase was purchased from Cell Signaling Technology (Danvers, MA, USA).

### Passive cutaneous anaphylaxis (PCA)

The mice were sensitised via the intravenous administration of 20 μg of anti-DNP-IgE. In the increased vascular permeability test, after 24 h, 0.6% DNFB was applied to the outside ear of each mouse, and 1% Evans blue (Sigma-Aldrich, St. Louis, MO, USA) was injected into the tail vein. One hour before DNFB application, 0.5%, 2% or 8% Eucalyptus oil was applied to the inside of the ear. After 30 min, the mice were sacrificed, and their ears were collected. Skin was excised and incubated with KOH at 55 °C for 24 h, and then, the extravasated Evans blue dye was extracted. The absorbance of the dye at 620 nm was measured using a multi-well spectrophotometer (Bio-Rad, Hercules, CA, USA).

In the ear swelling response test, no dye was administered, and Eucalyptus oil was applied to the inside of the ear. After 1 h, DNFB was applied to the outside of the ear, and the thickness of the ear was measured with a digital thickness gauge (Mitutoyo, Kanagawa, Japan).

### Preparation and culture of BMMCs

Bone marrow was collected from the femurs and tibias of wild-type BALB/c mice (female, 6–10 weeks old; Japan SLC, Shizuoka, Japan) after sacrifice via cervical dislocation. Their bone marrow cells were cultured in RPMI 1640 medium supplemented with 10% heat-inactivated FCS, 100 U/mL penicillin, 0.1 mg/mL streptomycin, 50 µM 2-ME and 5 ng/mL IL-3 (PeproTech, Cranbury, NJ, USA). The medium was changed every 3 days during culture. Mature BMMCs were obtained after 4 weeks of culture and stained with toluidine blue. Most (> 90%) of these cells expressed both c-Kit and FcεRI. All experiments were performed with BMMCs cultured for 4–7 weeks.

### β-Hexosaminidase release assay

BMMCs (1 × 10^5^ cells/mL) were incubated for 24 h with 1 μg/mL anti-DNP-IgE. Surface-bound IgE was cross-linked for 30 min at 37 °C using DNP-HSA. Reactions were stopped by placing the cells on ice. β-Hexosaminidase in the supernatant and cell lysate was incubated with 26 mM citrate buffer (pH 4.5) and 2.3 mg/mL p-nitrophenyl-N-acetyl-α-d-glucosaminide for 60 min at 37 °C. The reaction was developed by adding 0.4 M glycine (pH 10.7), and the absorbance was measured at 570 nm using a multi-plate reader (Bio-Rad). The percentage of β-hexosaminidase release was calculated relative to the total β-hexosaminidase content^22,40^.

### Histology

Ear specimens were fixed with 10% neutral buffered formalin and embedded in paraffin. Four-micrometre-thick sections cut from each paraffin block were stained routinely with H&E and toluidine blue. Extrusion of toluidine blue-stained granules was the evidence indicating mast cell degranulation. The number of mast cells and the percentage of degranulated mast cells at the dermal/epidermal junction were analysed in tissue sections, as described previously^24,25^.

### Cell viability assay

BMMCs (1 × 10^4^ cells/mL) were grown in 96-well micro-titre plates for 24 h. Eucalyptus oil or 1,8-cineole were added to the cells at a concentration of 0.5, 1.0, 5.0, 10.0 or 50.0 μg/mL, followed by incubation for 24 h. The cytotoxic effects of Eucalyptus oil and 1,8-cineole were evaluated using the conventional 3-(4,5-dimethylthiazol-2-yl)-2,5-diphenyltetrazolium bromide (MTT) assay. Specifically, 20 μL of MTT (5 mg/mL) was added to each well, followed by incubation at 37 °C for 4 h. The medium was removed, and 200 μL of DMSO was added to each well. The optical density was measured at 490 nm using a multi-well spectrophotometer (Bio-Rad).

### Measurement of intracellular calcium levels

Intracellular calcium levels were measured using a Calcium kit Fluo 4 (Dojindo Laboratories, Kumamoto, Japan) according to the manufacturer’s instructions^45,46^. BMMCs were seeded at 4.0 × 10^4^ cells/well in a 96-well black culture plate and sensitised with anti-DNP IgE (50 ng/mL) at 37 °C overnight. After washing with PBS, the cells were incubated with Fluo 4-AM for 1 h at 37 °C. Cells were again washed with PBS, treated with the recording buffer containing Eucalyptus oil and 1,8-cineole, and then incubated at 37 °C for 30 min. Next, the cells were stimulated with DNP-HSA (50 ng/mL), and the fluorescence intensity was immediately monitored with an excitation wavelength of 485 nm and an emission wavelength of 535 nm using a microplate reader (BioTeh, Winooski, St, USA).

### ELISA for histamine, IL-4, IL-13, prostaglandin D2 and leukotriene C4 production

BMMCs were stimulated as described previously. The cell culture supernatants were harvested 3 h after stimulation, and IL-6 and IL-13 concentrations were measured using ELISA kits (R&D Systems, Minneapolis, MN, USA). The prostaglandin D2 and leukotriene C4 concentrations in cell culture supernatants were measured at 1 h after stimulation using ELISA kits (Cayman Chemical, Ann Arbor, MI, USA) according to the manufacturer’s instructions. Histamine concentrations in cell culture supernatants were measured at 30 min after stimulation using an ELISA kit (Cayman Chemical) according to the manufacturer’s instructions.

### Immunoblot analysis

BMMCs were washed and lysed with iced lysis buffer (1% Triton X-100, 50 mM Tris–HCl [pH 7.5], 150 mM NaCl, protease inhibitor cocktail and phosphatase inhibitor cocktail; Nacalai Tesque)^47^. The lysates were subjected to SDS-PAGE and immunoblotting. The reactive bands were visualised using a 5-bromo-4-chloro-3-indolyl phosphate/NBT colour development substrate (Promega, Madison, WI, USA) or an ECL chemiluminescence detection kit. Densitometric analysis of the data obtained from at least three independent experiments was performed using a cooled CCD camera system EZ-Capture II and CS analyser ver. 3.00 software (ATTO, Tokyo, Japan).

### Statistical analysis

All data are expressed as the mean ± SEM. Each experiment was performed independently at least three times. For statistical significance, two-tailed Student’s *t*-test was performed when the results were between two groups. Comparisons between other groups were performed by one-way analysis of variance using Tukey–Kramer or Dunnett’s multiple comparison test.

### Ethical approval

All animal testing protocols were approved by the Animal Experiment Committee of Ikeda Mohando Co., Ltd. The Animal Experiment Committee of Ikeda Mohando Co., Ltd., has been certified by the Japan Health Science Foundation, including on-site surveys.

## Supplementary information

Supplementary Information
