# Supplementary material for: Eucalyptus oil reduces allergic reactions and suppresses mast cell degranulation by downregulating IgE-FcεRI signalling
Source: Sci Rep. 2020 Dec 1;10:20940. doi: 10.1038/s41598-020-77039-5 (PMC7708995; doi:10.1038/s41598-020-77039-5)
Supplement: Supplementary file 1 — Supplementary Information [file 41598_2020_77039_MOESM1_ESM.pdf]

Supplementary Information

Eucalyptus oil reduces allergic reactions and suppresses mast cell degranulation by downregulating IgE-FcεRI signalling

Tomoya Nakamura, Naoki Yoshida, Yu Yamanoi, Akira Honryo, Hiroyuki Tomita, Yoshihiko Kojima

Department of R&D Center, Ikeda Mohando Co., Ltd, Toyama, Japan

Correspondence: Tomoya Nakamura
Department of R&D center, Ikeda Mohando Co., Ltd.
16 Jinden, Kamiichi, Nakaniikawa, Toyama, Japan 930-0394
Telephone: +81-76-472-6411
Fax: +81-76-472-2277
E-mail: nakamura.t@ikedamohando.co.jp

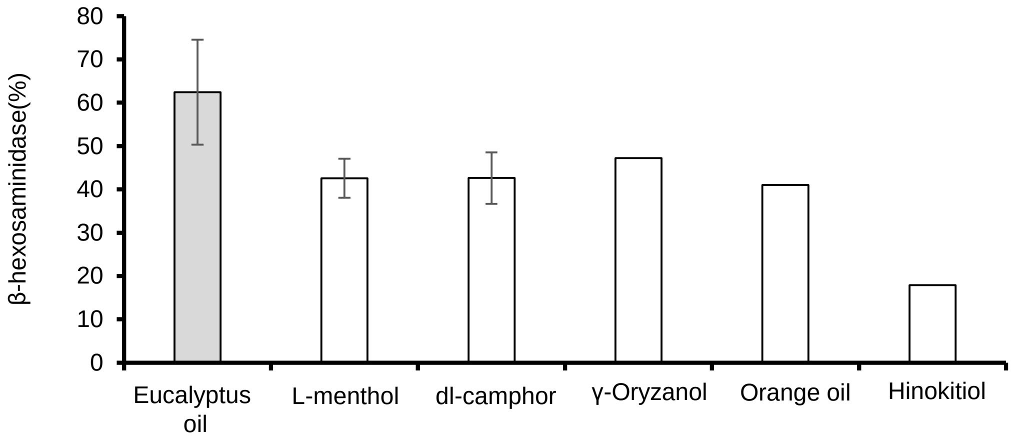

Supplementary Figure 1.
Screening of plant oil in degranulation inhibitory effect test of RBL-2H3 cells. β-HEX in RBL-2H3 cells. β-HEX valuesare expressed as percentage of total β-HEX present in the corresponding cell lysate.
Eucalyptus oil,L-menthol,dl-camphor: Data shown are means± SEM (N=3)
γ-Oryzanol, Orange oil, hinokitiol: Data shown are means (N=2)
Eucalyptus oil, L-menthol, dl-camphor and Orange oil were purchased from Nippon Terpene Chemicals.
Hinokitiol was purchased from Wako.
γ-Oryzanol was purchased from Tsuno.

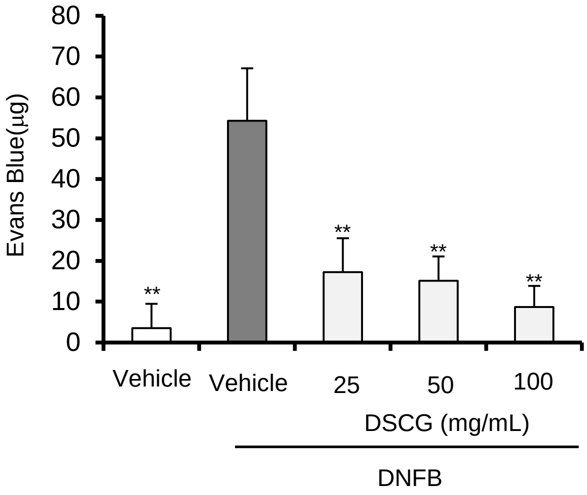

Supplementary Figure 2

Anti-DNP-IgE was injected intravenously 24 h before DNFB application. One hour before DNFB application, DSCG (sodium cromoglicate) or vehicle (PBS) was applied to the inside of each ear of all animals. DNFB was applied to the outside of the ear, followed by an immediate injection of Evans blue dye. Thirty minutes later, the ears were excised, and the absorbance of the dye was measured. Data are presented as the mean ± SEM (N = 6/group). The statistical significance of the differences was assessed using Dunnett's test (***P* < 0.01 versus vehicle-DNFB).

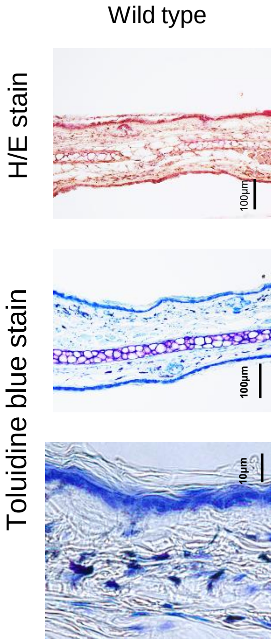

Supplementary Figure 3.
Haematoxylin and eosin-stained and toluidine blue-stained images ofear tissue in WBB6F1+/+ (wild-type) mouse.

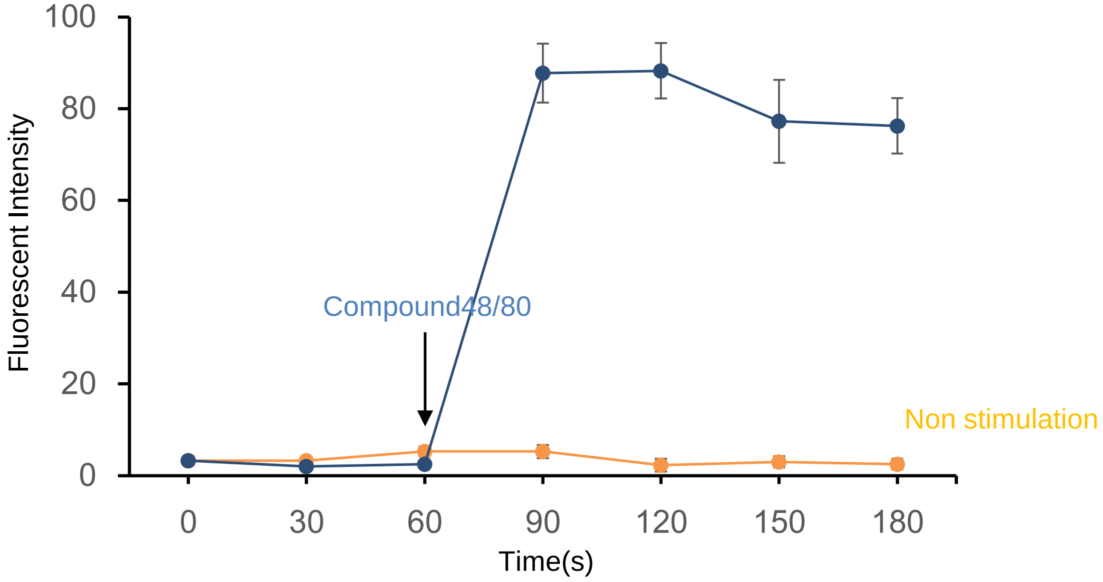

Supplementary Figure 4
Time course of intracellular Ca^{2+} concentration in BMMC. BMMCs were incubated in medium containing Fluo 4-AM. BMMCs were stimulated with Comp48/80 60s after the start of monitoring the fluorescence intensity. Data are displayed as mean ± SEM (N = 5).
Concentration of Compound 48/80 was as reported by Rothschild,A.M.(1970)
Rothschild,A.M. Mechanisms of histamine release by compound 48/80. Br J Pharmacol. 38.253-62 (1970)

Lyn:56kDa

100kDa-
75kDa-
50kDa-

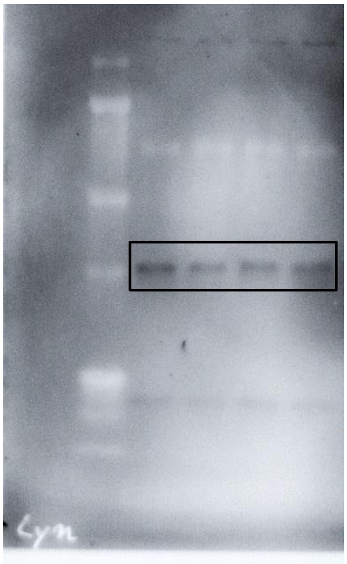

DNP-HSA(ng/ml)	0	0	50	50
1,8-Cineol (μg/ml)	0	0.5	0	0.5

p-Lyn:56kDa

100kDa-
75kDa-
50kDa-

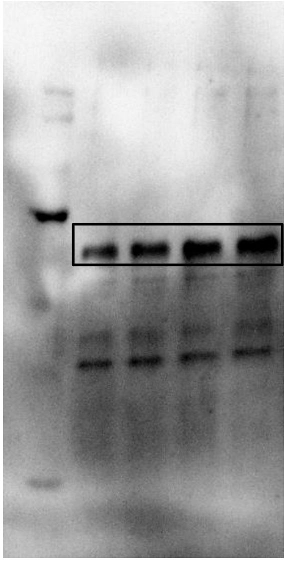

DNP-HSA(ng/ml)	0	0	50	50
1,8-Cineol (μg/ml)	0	0.5	0	0.5

Syk:72kDa

100kDa-
75kDa-
50kDa-

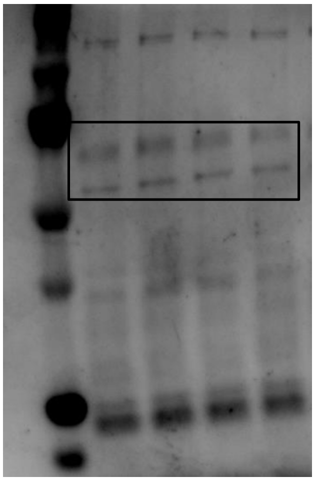

DNP-HSA(ng/ml)	0	0	50	50
1,8-Cineol (μg/ml)	0	0.5	0	0.5

p-Syk:72kDa

100kDa-
75kDa-
50kDa-

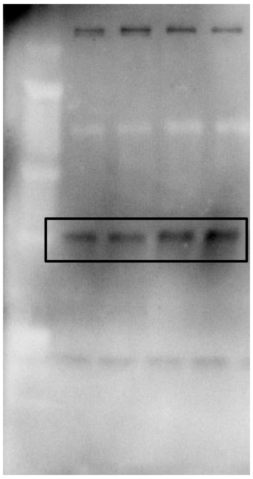

DNP-HSA(ng/ml)	0	0	50	50
1,8-Cineol (μg/ml)	0	0.5	0	0.5

Supplementary figure5. Uncropped immunoblot used in Figure 6. (Effect of 1,8-cineol on the phosphorylation of Syk and Lyn.)

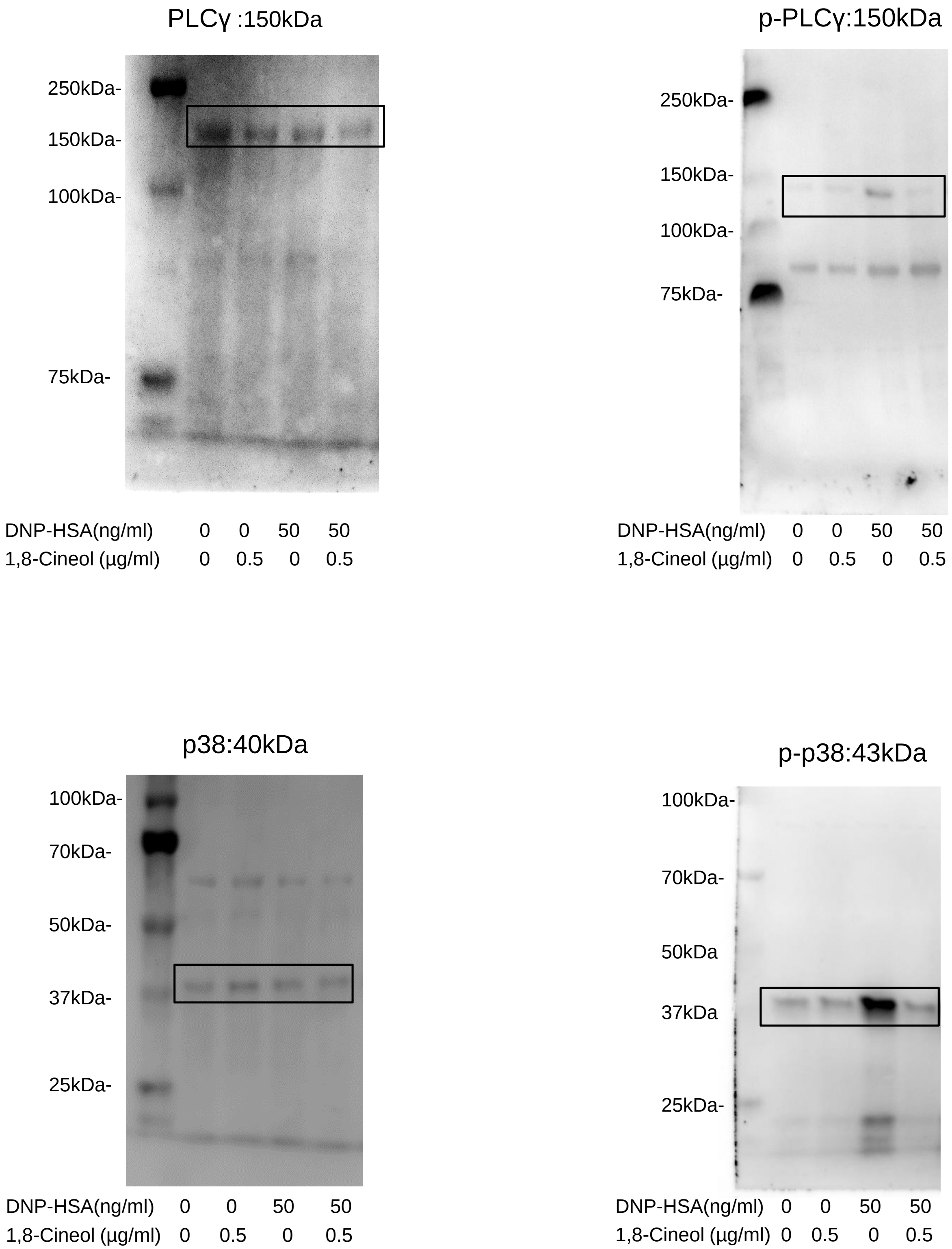

Supplementary figure6. Uncropped immunoblot used in Fig.7 a,b (Effect of 1,8-cineol on the phosphorylation of PLCγ, and p38)

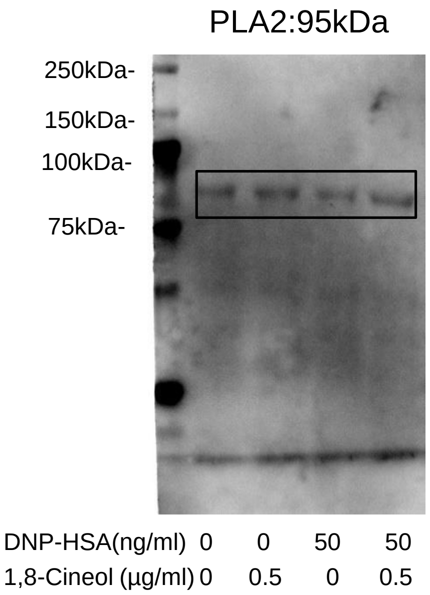

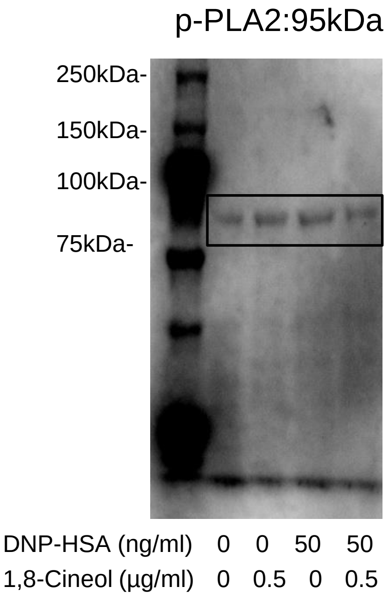

Supplementary figure7. Uncropped immunoblot used in Fig.7 c (Effect of 1,8-cineol on the phosphorylation of PLA2)
